# A spinoglenoid cyst compressing on the suprascapular nerve causing supraspinatus and infraspinatus muscle weakness: A case report

**DOI:** 10.1016/j.ijscr.2020.04.001

**Published:** 2020-05-15

**Authors:** Joseph Maalouly, Dany Aouad, Antonios Tawk, Georges El Rassi

**Affiliations:** a Department of Orthopedic Surgery and Traumatology Saint Georges University Medical Center, Balamand University, P.O. Box 166378, Achrafieh, Beirut, 1100 2807, Lebanon; b St Georges University Medical Center, Beirut, Achrafieh, St Georges Street, Lebanon

**Keywords:** Spinoglenoid cyst, Shoulder, Arthroscopy

## Abstract

**Introduction:**

Spinoglenoid cysts are a rare cause of suprascapular neuropathy of compressive etiology in which the patient may present with shoulder pain and muscle hypotrophy. MRIs are the imaging modality of choice showing location, size, and extent of the lesion for preoperative assessment. Currently, surgical intervention is the preferred approach for the management of a spinoglenoid cyst with arthroscopy being favored over the open technique.

**Case presentation:**

A 25-year-old male patient presenting with right posterior shoulder pain and decreased range of motion associated with the development of progressive weakness of the supraspinatus and infraspinatus muscles. Right shoulder MRI showed a posterior tear of the glenoid labrum and a spinoglenoid ganglion cyst. Preoperative electroneuromyography was suggestive of a suprascapular nerve impairment.

**Follow-up:**

Six months postoperatively, the patient has regained full range of motion of his right shoulder with no residue signs of muscle hypotrophy.

**Conclusion:**

Spinoglenoid cysts can cause compressive neuropathy with associated pain and muscle hypotrophy. MRI and EMG can help in establishing the diagnosis and in the pre-operative assessment. Surgical intervention with arthroscopy is currently the technique preferred by most surgeons.

## Highlights

• A case of arthroscopically treated spinoglenoid cyst in a young athlete compressing on the suprascapular nerve causing supra/infraspinatus weakness.
• Treatment consisted of repairing the labral tear leading to the cyst instead of drainage, leading to a lower rate of recurrence, with self resorption.
• The suprascapular nerve compression has resolved without surgically mobilizing the nerve itself.
• MRI and EMG can help in establishing the diagnosis and in the pre-operative assessment.

## 1 Introduction

A wide variety of pathologies may be implicated in the manifestation of neuropathies of the upper extremities. Unfortunately, the diagnosis may be delayed for several months in many cases of upper extremities neuropathies [1,2]. Kopell and Thompson (1959) were the first to describe a suprascapular neuropathy of compressive etiology as a pathological entity to be considered as part of the differential diagnosis in patients presenting with shoulder pain, decreased muscle mass (isolated infraspinatus muscle atrophy), and lateral rotation weakness [1,3,4]. Magnetic resonance imaging (MRI) has become the imaging modality of choice used in the diagnosis of the etiology of shoulder pain. Subsequently, there was an increase in the frequency of identification of shoulder cyst as the cause of shoulder pain and neuropathy [[5], [6], [7]]. Moreover, MRI allows the identification of the location, and size of the cyst as well as the presence of any concomitant pathology such as labral tears. Despite its false negative rate, electroneuromyography (EMG) can also be used in addition to MRI to establish the diagnosis of a compressive spinoglenoid cyst as an etiology of the upper extremity neuropathy. A spinoglenoid cyst in the spinoglenoid notch or even in the suprascapular notch can cause a compressive suprascapular neuropathy [8]. The literature reports that a spinoglenoid notch cyst may develop secondary to SLAP type II lesion as the labral tear forms a one-way valve allowing the synovial fluid to extravasate and accumulate outside the shoulder joint [[9], [10], [11]]. The typical presentation of a patient with a spinoglenoid cyst compressing on the suprascapular nerve is vague shoulder pain along with infraspinatus muscle weakness. However, should the spinoglenoid cyst be large enough; supraspinatus muscle weakness may also be present [12]. Several approaches have been proposed for the management of a symptomatic spinoglenoid cyst including observation [5,8], needle aspiration under ultrasound (US) or computed tomography (CT) guidance, or surgical intervention [5,13]. The surgical intervention can range from open drainage of the cyst to arthroscopic procedure involving either/both the cyst or/and the labrum of the glenoid [5,8,14,15]. Currently, surgical intervention is the preferred approach by most surgeons not only due to the unresponsiveness in nonoperative management, but also due to worsening of the symptoms of the patient. Moreover, the literature shows an increased risk of cyst recurrence after needle aspiration [5,12,16]. As for the surgical approach, arthroscopy is favored over the open technique since it is less invasive and it addresses any potential concomitant intraarticular pathology [5,17,18]. The literature describes several arthroscopic modalities for the treatment of a spinoglenoid cyst such as labrum repair without cyst resection, labrum repair with intraarticular arthroscopic cyst decompression, and cyst decompression through the subacromial space [5,[12], [13], [14],^19^,^20^]. However, the literature still does not contain a clear agreement on which arthroscopic technique prevails over the other [12].

Herein, we present a case of a patient presenting with posterior shoulder pain associated with supraspinatus and infraspinatus muscles weakness secondary to a spinoglenoid cyst compressing on the suprascapular nerve.

## 2 Case presentation

A 25-year-old male patient with a positive medical history for minor thalassemia and Gilbert syndrome presented to the hospital with a chief complaint of right posterior shoulder pain associated with weakness of the supraspinatus and infraspinatus muscle of gradual progression over the course of 1.5 years. The patient noted pain and difficulty with overhead activities; thus, interfering with his activities of daily living. The patient explained that his right shoulder pain is exacerbated upon abduction and external rotation. He denied any traumatic event sustained to the right shoulder.

**Fig. 1:**
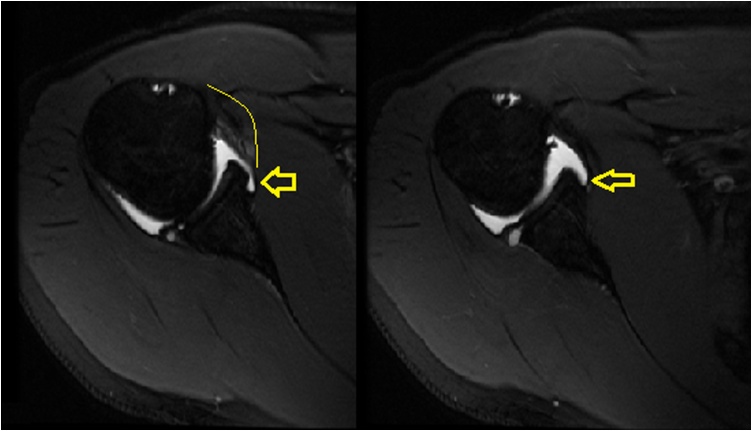
Axial cuts of right shoulder MRI showing posterior labral tear of the glenoid and cyst posteriorly.

**Fig. 2:**
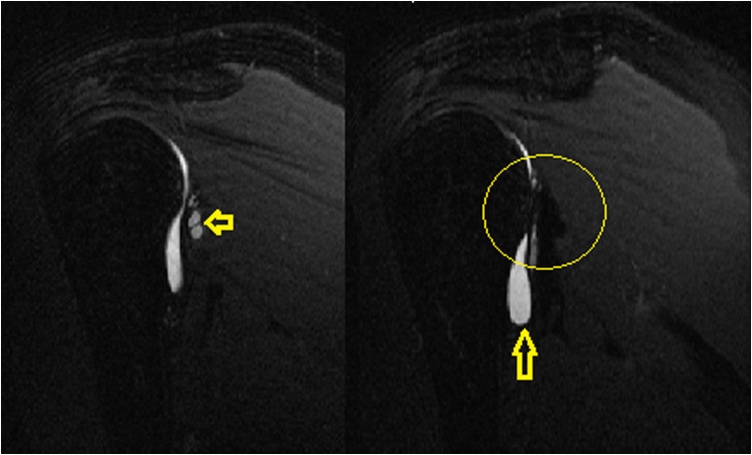
Sagittal MRI cuts of the right shoulder showing posterior cyst formation with the posterior labral tear of the glenoid.

**Fig. 3:**
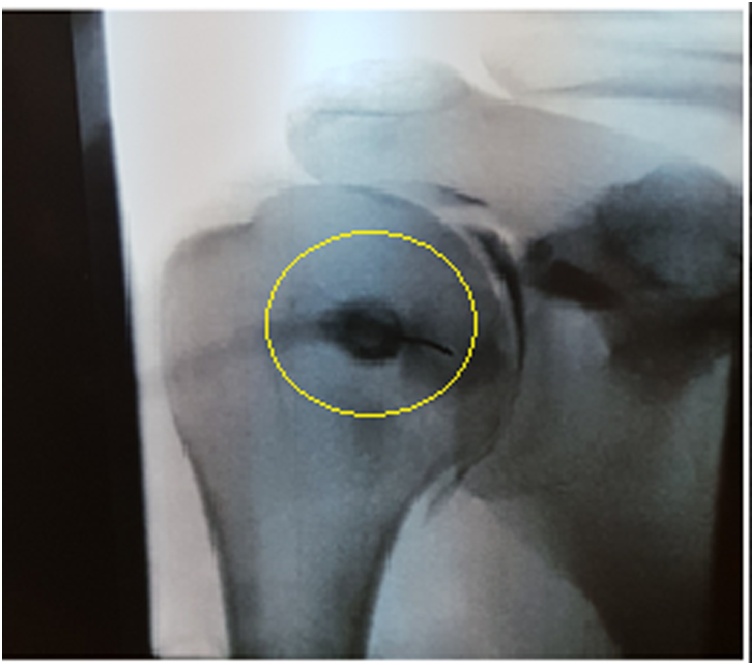
X-ray films under fluoroscopy with intraarticular contrast showing cyst and fluid extravasation.

On physical examination, there was unidirectional shoulder instability, and decreased range of motion associated with clinical evidence of weakness of the supraspinatus and infraspinatus muscles. The patient showed clinical evidence of muscle wasting or hypotrophy. Moreover, the patient had a positive O’Brien active compression test, 3+ anterior load shift, 2+ posterior load shift, and a positive relocation test. Moreover, the patient had a negative Hawkins’ test, Neer’s sign, empty can test, and inverted empty can test. Plain radiographs showed no bony abnormalities. Subsequently, an MRI was taken of the right shoulder revealing a posterior tear to the glenoid labrum and a spinoglenoid ganglion cyst (Fig. 1, Fig. 2). Fluoroscopy with contrast was also done, also confirming the diagnosis (Fig. 3). Preoperative electroneuromyography was suggestive of a suprascapular nerve impairment.

The patient was scheduled for right shoulder arthroscopy. Under general anesthesia, the patient’s right upper extremity was scrubbed and draped in beach chair position. The entry in the shoulder was by scope posterior portal while the insertion was under vision of anterior portal. Arthroscopic inspection confirmed MRI findings of posterior labral tear of the glenoid. The posterior-inferior labral tear is addressed. The 9 to 6 o’clock area of the glenoid rim is debrided with a burr, lasso suture is used to bring the labrum into place. Then two anchors were used to fix the tissue ensuring adequate capsule tension which led to cyst shrinkage.

At six month follow-up in the clinic as an outpatient, the patient denied any existing pain located at his right shoulder area or any associated muscle weakness interfering with his activities of daily living. Upon examination, the patient had regained full range of motion of his right glenohumeral joint without any limitations. The patient showed no residue signs of muscle hypotrophy and has regained the muscle mass which was previously hypotrophied upon his initial presentation.

## 3 Discussion

Compressive neuropathy of the suprascapular nerve is a rare pathological entity responsible for pain and functional abnormalities of the shoulder. Reports in the literature state that a spinoglenoid cyst is associated with a posterior labral tear. It is postulated that the labral tear acts as a one-way valve allowing the extravasation of the synovial fluid into the extra-articular space without allowing the return of this fluid [1]. Hence, an indirect arthroscopic decompression of the spinoglenoid cyst involve the repair of the labral tear with close observation for the resolution of the cyst. This has shown decrease in the postoperative recurrence risk of spinoglenoid cyst.

Although more than 50% of the associated glenoid labral tears are missed, MRI remains the gold standard for establishing the diagnosis of a spinoglenoid cyst [1,14]. An alternative imaging modality used for the preoperative assessment when there is high clinical suspicion of a spinoglenoid cyst is ultrasound [7,21]. Since patients with spinoglenoid cysts may present with muscular atrophy, electromyography (EMG) is an essential diagnostic modality to assess the presence of any associated neurologic involvement responsible for the muscular deficit. An electromyography helps to differentiate if the compressive effect of the spinoglenoid cyst is on the supraspinatus and the infraspinatus or the infraspinatus only [1].

Open drainage of a spinoglenoid cyst has shown high rates of recurrence postoperatively since the associated posterior labral tear is not dealt with. On the other hand, arthroscopic drainage of the cyst can be direct or indirect with the possibility of repairing the associated posterior labral tear. Moreover, arthroscopic drainage is associated with lower morbidity [1,14].

In the above presented case, the spinoglenoid cyst has not been directly drained, however the labral tear has been repaired using suture anchors.. Connection between the intra-labral space and the cyst itself has been obliterated, which lead the cyst to self-resorb over time. Hence, in such cases, labral tears repair is of high importance without cyst drainage, decreasing the risk of recurrence.

## 4 Conclusion

Spinoglenoid cysts account for a rare etiology of compressive neuropathy of the suprascapular nerve in which patients may present with shoulder pain and muscle hypotrophy. MRI and electroneuromyography help in establishing the diagnosis, and in the preoperative assessment of the extent of the lesion. Arthroscopic drainage of the spinoglenoid cyst was shown to be superior to open drainage approach.

## Declaration of competing interest

The authors declare no conflict of interest regarding the publication of this article.

## Funding

No funds were received in support of this study.

## Ethical approval

Ethics committee has given approval for publication.

## Consent

Written informed consent was obtained from the patient for publication of this case report and accompanying images. A copy of the written consent is available for review by the Editor-in-Chief of this journal on request.

No identity identifiers are present whatsoever in the manuscript.

## Author contribution

Joseph Maalouly: contributed to the writing and editing of this article.

Antonios Tawk: contributed to the writing and referencing of this article.

Dany Aouad: contributed to the writing of this article and the submission process.

Georges El Rassi: Surgeon who presented this case, writing and editing of the article.

## Registration of research studies

This case has been registered in the IRB committee of St Georges Hospital University Hospital.

## Guarantor

Dr Georges El Rassi.
