# Supplementary material for: A spinoglenoid cyst compressing on the suprascapular nerve causing supraspinatus and infraspinatus muscle weakness: A case report
Source: Int J Surg Case Rep. 2020 May 15;71:266–9. doi: 10.1016/j.ijscr.2020.04.001 (PMC7264009; doi:10.1016/j.ijscr.2020.04.001)
Supplement: Supplementary file 2 [file mmc2.docx]

**Video Legend:** Arthroscopic posterior labral tear repair. Starting with shaving of the posterior labral tear under direct vision. An awl is used to insert the drill in order to prepare for placement of the anchor at the required site. The anchor is then placed along with two sutures, which will be used to repair the posterior labral tear. Similarly, Preparation of second anchor placement in a more posterior fashion. The second anchor is placed, and suture of the labrum is done under direct vision with the help of lasso sutures. Satisfactory repair results seen with full coverage over the cyst, obliterating its connection with the intra-articular space.
